# Crucial Role for Early Growth Response-1 in the Transcriptional Regulation of miR-20b in Breast Cancer

**DOI:** 10.18632/oncotarget.1165

**Published:** 2013-07-28

**Authors:** Dongping Li, Yaroslav Ilnytskyy, Anna Kovalchuk, Levon M. Khachigian, Roderick T. Bronson, Bo Wang, Olga Kovalchuk

**Affiliations:** ^1^ Department of Biological Sciences, University of Lethbridge, Lethbridge, Canada; ^2^ Department of Biochemistry, Qiqihar Medical University, Qiqihar, P.R. China; ^3^ Centre for Vascular Research, University of New South Wales, Sydney, Australia; ^4^ The Dana Farber/Harvard Comprehensive Cancer Center, Boston, Massachusetts, USA

**Keywords:** EGR1, miR-20b, transcription, PTEN, BRCA1, breast cancer, proliferation, migration, cell cycle arrest

## Abstract

Transcriptional regulation of miRNAs that control the pathogenesis of breast cancer remains largely unknown. Here, we showed that ionizing radiation, a known breast carcinogen, triggered the differential expression of miR-20b in mammary tissues. We identified several GC-rich consensus binding motifs for the zinc finger transcription factor early growth response-1 (EGR1) in miR-20b promoter. miR-20b was upregulated by IR and its upregulation correlated with EGR1 expression in the breast cancer cell line HCC1806. Therefore, we used HCC1806 cells as a model system to explore the role of EGR1 in miR-20b transcription. siRNA knockdown of EGR1 attenuated miR-20b expression. Luciferase assays showed that whereas EGR1 stimulated luciferase activity driven by the wild-type miR-20b promoter, this induction was abolished in the mutant miR-20 promoter construct. We noted significant enrichment of EGR1 at miR-20b promoter in HCC1806 cells compared with normal human mammary epithelial cells. Suppression of miR-20b significantly inhibited HCC1806 cell proliferation and migration, and led to G 0/G 1 and S phase arrest. *In vitro* RNA-pull down assays indicated that miR-20b targets numerous tumor suppressors, including PTEN and BRCA1, which were downregulated in HCC1806. Conversely, suppression of miR-20b increased PTEN and BRCA1 levels. Moreover, immunohistochemical and FISH analyses showed that the miR-20b expression correlated significantly with EGR1 levels in breast cancer tissues. Our findings thus demonstrate for the first time that EGR1 is a key player in the transcriptional control of miR-20b, and miR-20b may in turn function as an oncogene by contributing to breast tumorigenesis via tumor suppressor targeting.

## INTRODUCTION

Breast cancer is the most common malignancy in women worldwide and the second leading cause of cancer-related deaths among North American women[1]. Most breast cancer patients undergo radiation diagnosis and are treated with radiotherapy. In addition to being an important treatment modality, ionizing radiation (IR) is a potent tumor-causing agent that has been linked to breast cancer development[2-4]. However, the exact molecular etiology of IR-induced mammary gland carcinogenesis remains unknown. Breast cancer is currently recognized as a genetic and an epigenetic disease[5]. The contribution of epigenetic alterations to breast carcinogenesis remains relatively obscure.

In recent years, one of the key advances in our understanding of the fundamental mechanisms of gene regulation has been the discovery of microRNAs (miRNAs/miRs). miRNAs are small noncoding RNA molecules that regulate gene expression either through translational repression or mRNA degradation, as determined by the degree of complementarity to the 3' untranslated regions of cognate mRNAs[6, 7]. Approximately 30% of all protein-coding genes are assumed to be targets of miRNAs[8]. miRNAs possess diverse functions in many biologic and pathologic processes, including control of cell differentiation, proliferation, and apoptosis. Aberrant expression and dysregulation of miRNAs contribute to tumorigenesis, angiogenesis, and metastasis[6,9,10]. Current evidence indicates that miRNAs can serve as either tumor suppressors or oncogenes[11, 12].

miRNA also partake in genotoxic stress responses. Many genotoxic carcinogens affect miRNA patterns in the exposed tissues and organs. Amongst those, IR profoundly affects tissue miRNAome. Recently we have shown that IR exposure affects mammary gland tissue and causes profound deregulation of miRNA expression. Amongst miRNAs, miR-20b was significantly affected.

miRNA-20b (miR-20b) is encoded by the miR-106a-363 cluster which, together with the miR-17-92 and miR-106b-25 clusters, forms a large family of highly similar miRNAs called the miR-17 family[13]. Members of the miR-17 family are often upregulated in many human malignancies, such as lung cancer and leukemias[14-17]. Ectopic expression of miR-17 promotes motility and invasion of glioblastoma cells through targeting PTEN[18]. The high expression levels of miR-20b in gastric cancer constitute a negative survival prognostic factor[19]. miR-20b is upregulated in c-Myc-induced mouse mammary tumors[20]. Furthermore, several lines of evidence demonstrate that miR-20b downregulates ERα; (estrogen receptor alpha) [21] and modulates VEGF expression by targeting HIF-1α and STAT3[22] in MCF7 breast cancer cells.

Transcriptional regulation of miR-20b in human cancers remains poorly understood to date. We therefore explored the transcription factor(s) involved in miR-20b expression and the role of miR-20b in breast tumorigenesis. The data presented in this paper indicate that IR induces miR-20b expression in rat mammary gland tissues in a dose- and time-dependent manner. We also show that miR-20b is upregulated in HCC1806 breast cancer cells, and this upregulation correlates with EGR1 expression. We provide evidence that EGR1 controls miR-20b transcription via putative EGR1 binding motifs present in miR-20b promoter. Suppression of miR-20b inhibits HCC1806 proliferation and migration, resulting in G_0_/G_1_ and S phase arrest. Furthermore, we provide the key evidence that miR-20b targets tumor suppressors BRCA1 and PTEN. Finally, immunohistochemical and FISH analyses indicate that miR-20b is elevated in 30% of breast cancer and 50% of metastatic breast cancer specimens examined, and this upregulation correlates significantly with EGR1 levels.

## RESULTS

### IR-induced miR-20b expression in mammary gland tissues and cells

**Figure 1:**
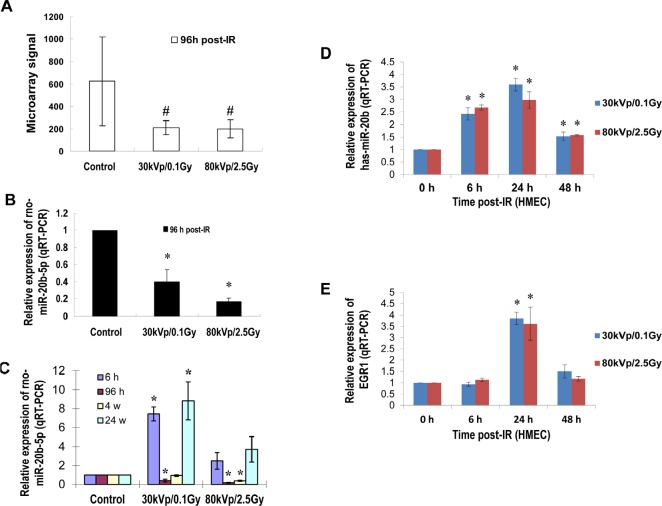
IR induces miR-20b expression in mammary gland tissues/cells in a dose- and time-dependent manner (A and B) Total RNA isolated from the mammary gland tissues of six-week-old female Long Evans rats exposed to either 30 kVp/0.1 Gy, 80 kVp/2.5 Gy X-ray, or sham-treatment 96 hours post-irradiation was subjected to microRNA microarray; the levels of rno-miR-20b were determined by real-time RT-PCR. (C) Total RNA was isolated from the mammary gland tissues of six-week-old female Long Evans rats at different time points post-IR, and the levels of rno-miR-20b were examined by real-time RT-PCR. (D and E) Total RNA isolated from HMEC exposed to either 30 kVp/0.1 Gy or 80 kVp/2.5 Gy X-ray was subjected to real-time RT-PCR using primers for hsa-miR-20b and EGR1. The hash indicates *p*<0.1; the asterisk indicates *p*<0.05.

Our previous studies demonstrated that IR triggered a significant and sex-specific deregulation of the microRNAome, as well as altered levels of Dicer and components of the RNA-induced silencing complex in the spleen of C57BL/6 mice [23]. To understand the microRNAs that are differentially expressed in mammary gland tissues in response to IR, six-week old female Long Evans rats were exposed to different doses/energy X-ray and sacrificed at different time points after irradiation. microRNA microarray analysis showed that 96 hours after irradiation, miR-20b was significantly reduced (Fig. 1A). This result was confirmed by quantitative real-time RT-PCR (qRT-PCR, Fig. 1B). A similar response was also displayed in human mammary epithelial cells (HMEC) 96 hours post irradiation (Fig. S1A). The qRT-PCR using RNA samples from IR-exposed mammary gland tissues at different time points showed both a time- and dose-dependent expression of miR-20b (Fig. 1C). IR also triggered a rapid and transient induction of miR-20b in HMEC cells which peaked at 24 hour post-IR (Fig. 1D and Fig. S1A), and correlated with the IR-inducible EGR-1 expression (Fig. 1E, Fig. S1B and C; correlation r=0.81926 in 30 kVp/0.1 Gy group; correlation r=0.68675 in 80 kVp/2.5 Gy group), although the EGR1 mRNA was not elevated at 6 hour post-IR. In consideration of the involvement of gene copy numbers in gene expression, we determined the changes in copy number in HMEC cells as a response to IR. However, our results showed that IR did not affect the copy number of miR-20b gene (Fig. S2), indicating the involvement of other mechanisms in the control of IR-inducible miR-20b transcription. Software-based bioinformatics analysis (Promoter 2.0 Prediction Server and Genomatix) identified several putative EGR1 binding motifs present in miR-20b promoter (Fig. 3A). We therefore hypothesized that EGR1 may play a role in miR-20b transcription.

### EGR1 contributes to the transcriptional regulation of miR-20b in breast cancer cells

**Figure 2:**
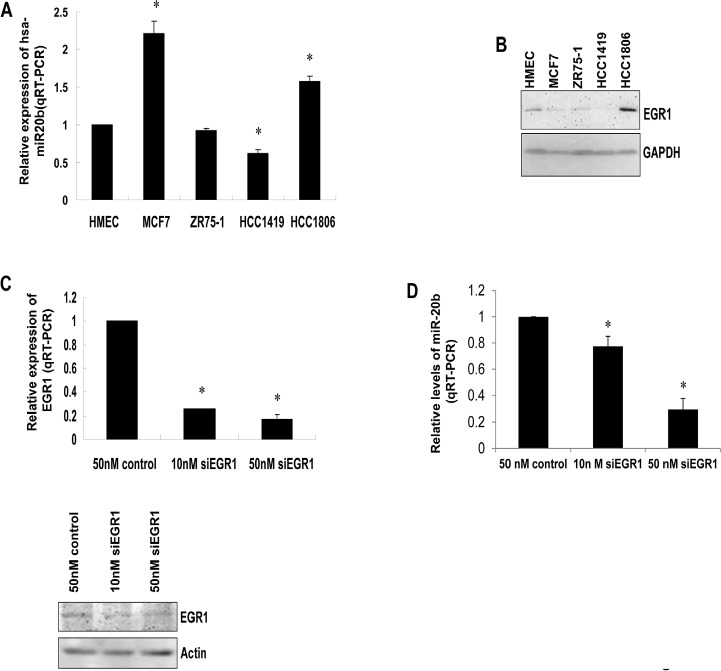
EGR1 correlates with miR-20b expression levels (A) Total RNA isolated from HMEC and breast cancer cell lines MCF7, ZR75-1, HCC1419, and HCC1806 was subjected to real-time RT-PCR with a primer set for miR-20b. (B) Whole cell lysates prepared from the above cell lines were subjected to Western blot analysis using antibodies against EGR1 and GAPDH. (C) HCC1806 cells were transiently transfected with either siEGR1 (siRNA targeting EGR1) or control siRNA; the levels of EGR1 mRNA and protein were determined by real-time RT-PCR (upper panel) and Western blot analysis (lower panel). (D) HCC1806 cells were transiently transfected with either siEGR1 or control siRNA; the levels of miR-20b were determined by real-time RT-PCR. The asterisk indicates *p*<0.05.

**Figure 3:**
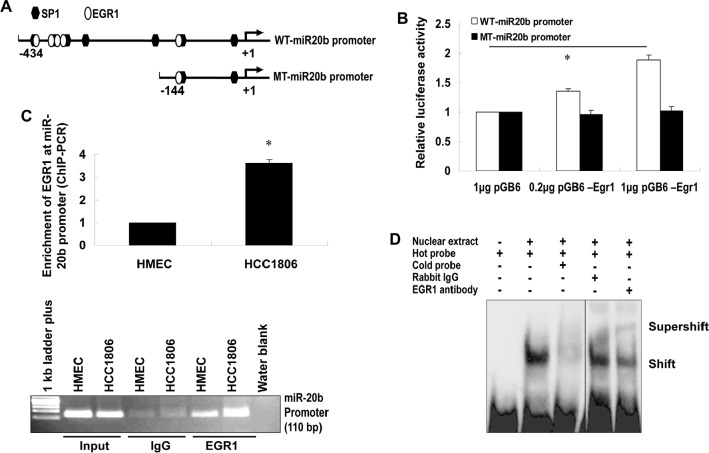
EGR1 regulates miR-20b transcription (A) The wild-type and mutant miR-20b promoter reporters used in this project. (B) HEK293 cells were transiently transfected with pGL3-WT-miR20b-Prom or pGL3-MT-miR20b-Prom and pCB6-Egr1 or pCB6; luciferase activity was detected according to the manufacturer's instruction. (C) Real-time ChIP-PCR and conventional ChIP-PCR were performed as described in “Materials and Methods”. (D) Nuclear extracts were prepared from HCC1806 cells, and EMSA was performed using ChIP-grade antibody to EGR1 according to the manufacturer's instruction. The asterisk indicates *p*<0.05.

To explore our hypothesis, we determined the expression of EGR1 and miR-20b, as well as the contribution of EGR1 to miR-20b transcription in breast cancer cells. qRT-PCR showed an aberrant expression of miR-20b in the breast cancer cell lines examined (Fig. 2A), which correlated with EGR1 expression (Fig. 2B), with the exception of MCF7. Knockdown of EGR1 in HCC1806 cells with the use of siEGR1 (siRNA targeting EGR1) resulted in a reduction in miR-20b expression (Fig. 2C and D). This reductioxn was particularly potent at the 50 nM siEGR1 dose (Fig. 2D). Ectopic expression of Egr1 caused induction in luciferase activity in a reporter construct harboring the wild-type miR-20b promoter in a dose-dependent fashion. This EGR1 responsiveness was completely abolished in the mutant construct (Fig. 3B). EGR1 was overexpressed in HCC1806 cells (Fig. 2B), and both real-time ChIP-PCR and conventional ChIP-PCR indicated that EGR1 was functionally enriched at miR-20b promoter in HCC1806 cells compared with normal HMEC (Fig. 3C). Furthermore, EMSA assays indicated that EGR1 specifically bound to miR-20b promoter (Fig. 3D). Taken together, these results suggested that EGR1 played a crucial role in controlling miR-20b transcription. We then determined the role of miR-20b in breast carcinogenesis.

### miR-20b is a key player in breast cancer cell proliferation, migration, and cell cycle control

**Figure 4:**
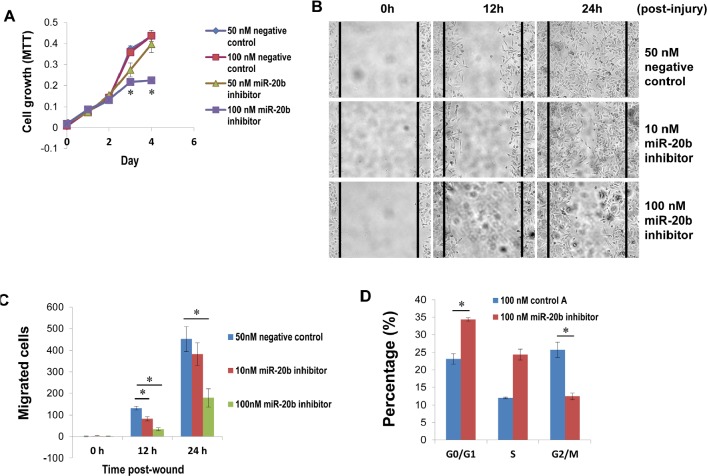
miR-20b inhibitor suppresses breast cancer cell proliferation and migration, as well as induces G_0_/G_1_ and S phase arrest (A) HCC1806 cells were transfected with either miR-20b inhibitor or a negative control; MTT assay (cell proliferation assay) was performed according to the manufacturer's instruction. (B and C) HCC1806 cells were transfected with either miR-20b inhibitor or negative control; 24 hours after transfection, wound-healing assay and statistical analysis of migrated cells were performed. (D) HCC1806 cells were transfected with either miR-20b inhibitor or negative control; 96 hours after transfection, cell cycle analysis was conducted using DB FACSCanto II Flow Cytometer. The asterisk indicates *p*<0.05.

**Figure 5:**
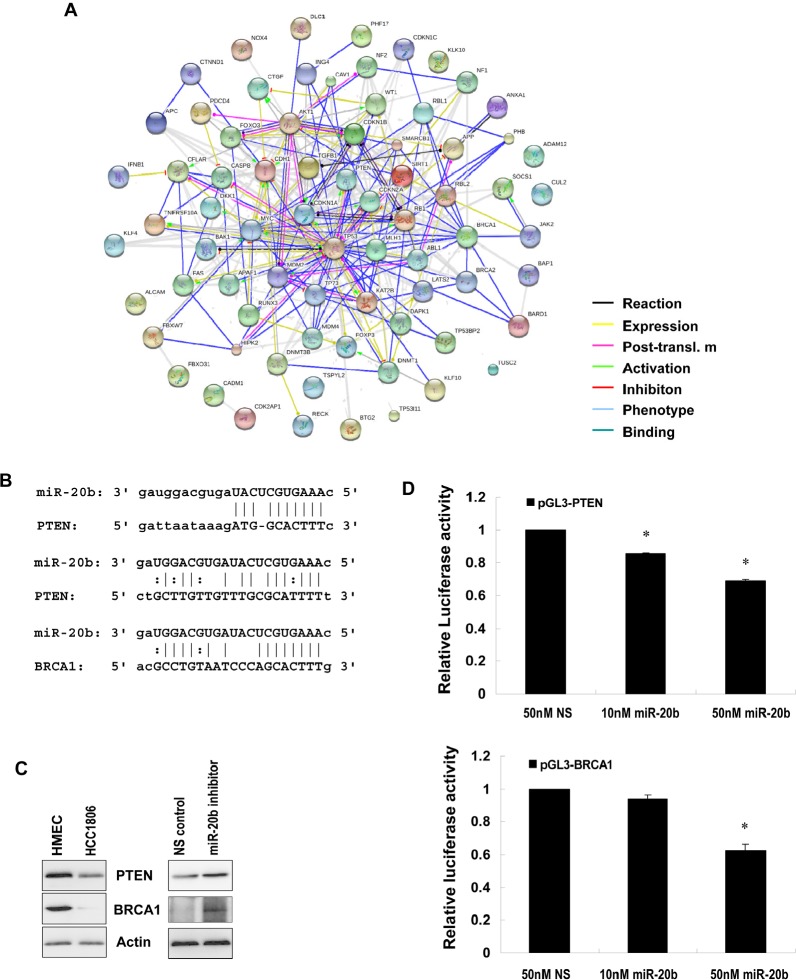
PTEN and BRCA1 are direct targets of miR-20b (A) The network of the predicted targets of hsa-miR-20b was generated using STRING 9.0. (B) Diagram of 3'UTR sequences of PTEN and BRCA1 targeted by hsa-miR-20b. (C) Whole cellular lysates prepared from HMEC, HCC1806, and HCC1806 transfected with either 50 nM miR-20b inhibitor or non-specific control for 72 hours were subjected to Western blot analysis using antibodies specific to PTEN and BRCA1. (D) HEK293 cells grown to 90% confluency were cotransfected with either pGL3-PTEN or pGL3-BRCA1 reporter, and the indicated concentration of hsa-miR-20b or 50 nM nonspecific miRNA as a control; 24 hours after transfection, luciferase activity was detected using Dual-Luciferase Reporter Assay System according to the manufacturer's instruction. The asterisk indicates *p*<0.05.

Because of the upregulation of miR-20b in HCC1806 cells, we selected this cell line as a model system to functionally suppress miR-20b with the use of specific inhibitors. HCC1806 cell proliferation was significantly suppressed by miR-20b inhibitor in an MTT assay (Fig. 4A), and HCC1806 cell migration was likewise inhibited in a wound healing assay (Fig. 4B and C). Inhibition of miR-20b also interestingly resulted in G_0_/G_1_ and S phase cell cycle arrest (Fig. 4D), although miR-20b inhibitor did not affect apoptosis (Fig. S3). To identify the target molecules of miR-20b that may be involved in these pathological processes, molecules that bind to miR-20b were pulled down in vitro and subjected to deep sequencing analysis. Software predictions by MIRANDA and RNAhybrid showed that miR-20b could bind to the 3' UTRs of many tumor suppressors (Fig. 5A) that are primarily associated with cell proliferation, invasion, apoptosis, and cell cycle control. Among the predicted targets of miR-20b, phosphatase and tensin homolog (PTEN, Fig. 5B) and breast cancer 1 gene (BRCA1, Fig. 5B) are critical in the maintenance of genomic stability, negative regulation of proliferative signaling, and prevention of cancer. Western blot analysis showed that PTEN and BRCA1 were downregulated in HCC1806 cells compared with HMEC, and that were inversely correlated with miR-20b expression in these cell lines (Fig. 5C, Fig. 2A). By contrast, suppression of miR-20b resulted in an elevation of the aforementioned proteins in HCC1806 cells (Fig. 5C). Luciferase activity in both pGL3-PTEN and pGL3-BRCA1 reporters was significantly reduced by miR-20b (Fig. 5D), suggesting that PTEN and BRCA1 were direct targets of miR-20b.

### EGR1 expression correlates with miR-20b expression in breast cancer specimens

**Figure 6:**
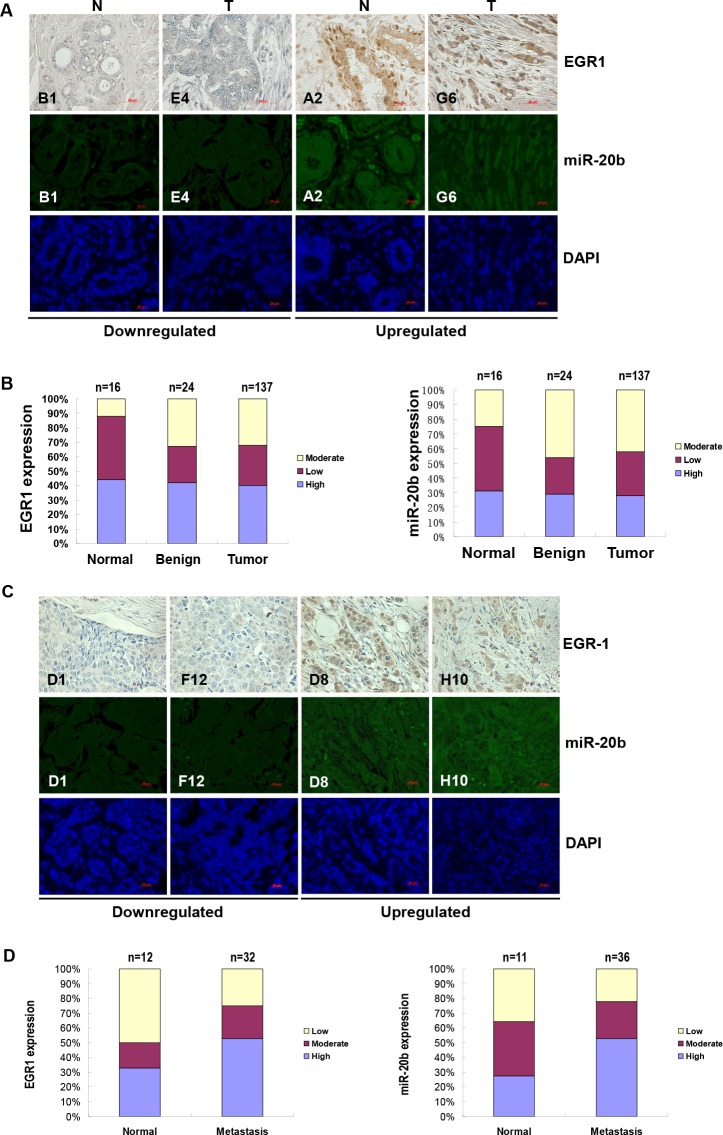
EGR1 expression is correlated with miR-20b expression in breast cancer tissues (A) Representatives of EGR1 and hsa-miR-20b stainings in the same sections of breast cancer tissue arrays. (B) Statistical and correlation analyses of EGR1 and hsa-miR-20b expression in breast cancer tissues. (C) Representatives of EGR1 and hsa-miR-20b staining in the same sections of metastatic breast cancer tissue arrays. (D) Statistical and correlation analyses of EGR1 and hsa-miR-20b expression in metastatic breast cancer tissues.

To further confirm the role of EGR1 in miR-20b transcription in diseased tissues, immunohistochemical staining and FISH analysis were performed to determine the expression of EGR1 and miR-20b in breast cancer tissue arrays. EGR1 was upregulated in 40% breast cancer tissues, and miR-20b was elevated in 30% breast cancer tissues examined: upregulated EGR1 correlated significantly with upregulated miR-20b in the normal, benign, and tumor tissues tested (Fig. 6A and B; r=0.99, p=0.032). Likewise, downregulation of EGR1 also correlated with downregulated miR-20b in the normal, benign, and tumor tissues examined (Fig. 6A and B; r=0.99, p=0.054). More important, EGR1 was overexpressed in 50% of the metastatic breast cancer tissues examined. Once again, this overexpresion correlated strongly with an upregulation of miR-20b (Fig. 6C and D). These results further confirm the role of EGR1 in the transcriptional control of miR-20b and that aberrant expression contributed to the development of breast cancer, particularly metastatic breast cancer.

### Expression of PTEN and BRCA1 is negatively correlated with miR-20b expression in metastatic breast cancers

To determine if the differentially expressed miR-20b contributes to the expression of PTEN and BRCA1 in breast cancer, we further looked at the levels of their expression in breast cancer cell lines and breast cancer tissue arrays. Western blot analysis showed that levels of PTEN and BRCA1 were downregulated in all breast cancer cell lines examined (Fig. S4). While only in MCF7 and HCC1806 lines (50%, n=4), the levels of PTEN and BRCA1 are negatively correlated with the miR-20b expression (Fig. 2A). Immunohistochemical analysis indicated that PTEN and BRCA1 are downregulated in 47% and 77.8% of metastatic breast cancer tissues, respectively (Fig. S5 and S6), and the levels of PTEN and BRCA1 were both negatively correlated with the miR-20b expression (Fig. 6D) (r=−0.996 and −0.778, respectively). Furthermore, the levels of PTEN and BRCA1 in normal and benign breast tissues were also negatively correlated with the miR-20b expression (Fig. S7 and S8, and Fig. 6B) (Normal: r=−0.949 and −0.749, respectively; benign: r=−0.761 and −0.52, respectively). However, no correlation was found in malignant breast cancer tissues. These results suggest that miR-20b contributes, at least in part, to the aberrant expression of PTEN and BRCA1 in breast cancer.

## DISCUSSION

In recent years, small noncoding RNAs, especially miRNAs, have been extensively investigated as possible key players in the process of breast cancer development and breast cancer treatment responses. However, all research efforts have primarily focused on identifying genes that miRNAs target and affect. Little is known about how miRNA transcription is regulated. A better understanding of the mechanisms that regulate miRNA transcription would provide an essential backdrop for future interventional approaches.

**Figure 7:**
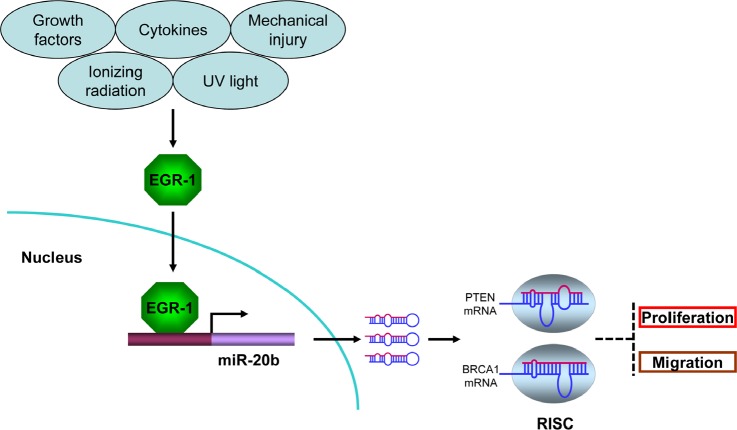
miR-20b transcriptionally activated by EGR1 directly targets PTEN and BRCA1 in breast cancer Serum-inducible zinc finger transcription factor EGR1 is induced and activated in response to a wide range of extracellular stimuli, including growth factors, cytokines, UV light, ionizing radiation, and mechanical injury. Once activated, EGR1 translocates into nucleus and binds to the consensus motifs at miR-20b promoter, leading to miR-20b transcription. The mature miR-20b assembles with other proteins to form RNA-induced silencing complex (RISC), the later recognizes and binds to PTEN and BRCA1 mRNAs, leading to either translational suppression or degradation of those two molecules, consequently resulting in breast cancer cell proliferation and migration.

This study for the first time demonstrated that EGR1 regulated miR-20b transcription and provided important clues on the role of miR-20b in breast tumorigenesis (Fig. 7). Breast cancer is a multifactorial and multistage process that involves many environmental and genetic factors. Among the environmental factors that cause breast cancer, IR may be one of the high-risk factors because it has been shown to strongly induce breast cancer in exposed individuals[2].Furthermore, the IR-induced mouse breast cancer model has been widely used in the field of breast cancer research. Unfortunately, the epigenetic mechanisms underlying IR-induced mammary carcinogenesis largely remain unknown. We showed in this paper that IR triggered miR-20b expression in mammary gland tissues in a dose- and time-dependent manner (Fig. 1C). Although IR is a putative inducer of genomic instability, including gene amplification [24], miR-20b gene copy number in our case did not contribute to IR-induced miR-20b expression (Fig. S1 and Fig. 1D).

Interestingly, bioinformatic analysis showed several putative EGR1 binding motifs present in miR-20b promoter, and EGR1 expression was correlated with miR-20b expression in HMEC cells in response to IR, suggesting a role of EGR1 in miR-20b transcription. Our deduction was confirmed by experiments performed on breast cancer cell lines, in which miR-20b expression was positively correlated with EGR1 expression in the cell lines examined, except for MCF7. Furthermore, knockdown of EGR1 resulted in a reduction in miR-20b expression. The regulation of gene expression is known as a complex process in which numerous mechanisms are involved, such as genetics, epigenetics and molecular biology, transcriptional and post-transcriptional levels. In MCF7, the EGR does not appear to contribute to the miR-20b transcription. That, however, may reflect the involvement of other transcription factors or mechanisms.

EGR1 is a zinc finger transcription factor that plays a crucial role in controlling cell growth, proliferation, differentiation and apoptosis [25-29]. EGR1 regulates transcription of target genes by binding to GC-rich consensus DNA elements present in the regulatory regions. EGR1 is induced in response to a wide range of extracellular stimuli that includes growth factors, cytokines, ionizing radiation, UV light, and mechanical injury [30-33]. Growing evidence indicates that EGR1 activation may serve as a key switch in many pathological processes, including cardiovascular disease and cancers. EGR1 has been indicated in the progression of breast, colon, prostate and esophageal cancers[34-38]. Elevated EGR1 in esophageal cancer plays an important role in mediating and maintaining growth-related oncogene/CXC chemokine receptor 2 proliferative signaling[34].EGR1 is overexpressed in primary human prostate carcinomas [36, 37], and several EGR1 target genes (*e.g.* insulin-like growth factor II, transforming growth factor β1, and platelet-derived growth factor A-chain) have been implicated in prostate tumorigenesis [37]. Knockdown of EGR1 suppresses prostate cancer cell proliferation and tumor development in transgenic adenocarcinoma mouse prostate mice [39]. Furthermore, DNAzymes targeting EGR1 inhibit breast cancer cell proliferation, migration and tumor growth in nude mice [38] although the underlying mechanisms are still unclear. Here, we provide evidence to show that miR-20b is a direct target of EGR1. Our findings demonstrate that EGR1 is associated with miR-20b expression in IR-exposed HMEC cells, breast cancer cell lines and tissues examined, and that EGR1 interacts with the miR-20b promoter and functionally regulates miR-20b transcription. Although here we only discuss the oncogenic role of EGR1, several lines of evidence have indicated a tumor suppressor role in both p53-dependent and –independent apoptosis [40, 41].

Although this is the first report regarding EGR1 in the transcriptional regulation of miR-20b, EGR1 was previously reported to regulate mir-106a expression[42]. This result suggested that in addition to the well-defined mRNA transcription, EGR1 may play a critical role in the transcriptional control of miRNAs of miR-17 family. The latters may be important in mediating the biological functions of EGR1.

The absence of ERα; in breast carcinomas has been known for years to be associated with a less-differentiated phenotype and with resistance to endocrine therapies, thus presenting poor prognosis. A recent study identified a new modulator of ERα;, miR-20b, which downregulated ERα; in MCF7 breast cancer cells [21]. However, the role of miR-20b in breast tumorigenesis remains elusive.

We showed here that miR-20b inhibitor dramatically suppressed HCC1806 breast cancer cell proliferation and migration resulting in a G_0_/G_1_ and S phase arrest in cell cycle that clearly indicated the key role of miR-20b in the development of this disease. To globally identify miR-20b targets associated with cell proliferation, migration, and cell cycle control, *in vitro* RNA pulldown and deep RNA sequencing analyses were performed. Our results indicated that miR-20b could target many tumor suppressors (Fig. 5A), including PTEN and BRCA1, which were of a particular interest to us (Fig. 5B), since the inhibitory role of these two genes in proliferation, migration, and cell cycle has been well documented [43-49]. PTEN is frequently mutated in human primary tumors and cell lines. The involvement of PTEN in human mammary tumorigenesis has been demonstrated from studies showing that germline PTEN mutations in Cowden disease predisposes afflicted individuals to breast cancer. The frequent loss of heterozygosity at the PTEN locus and reduced PTEN protein levels are often seen in sporadic breast cancers[43]. Germline mutation of BRCA1 frequently leads to hereditary breast and ovarian cancer (HBOC) syndrome, which accounts for 5% to 7% of all breast cancer cases. Individuals with HBOC syndrome have a 50% to 80% lifetime risk of developing breast cancer [44], suggesting the crucial role of loss-function of BRCA1 in the development of breast cancer. We showed in this paper that the expression of PTEN and BRCA1 was downregulated in HCC1806 breast cancer cells compared with HMEC cells, and this diminished presence was negatively correlated with miR-20b expression in such cell lines. Conversely, suppression of miR-20b with the use of its inhibitor remarkably enhanced the protein levels of PTEN and BRCA1 in HCC1806 cells (Fig. 5C). Furthermore, PTEN and BRCA1 are both downregulated in metastatic breast cancer tissues (Fig. S5 and S6), and that is negatively correlated with miR-2b expression in these tissues (Fig. 6D). Moreover, luciferase assays confirmed that PTEN and BRCA1 3' UTRs were direct targets of miR-20b (Fig. 5D). The expression of PTEN and BRCA1 in ZR75-1 and HCC1419 breast cancer cells and malignant breast cancer tissues (Fig. S4, S7 and S8), however, was not negatively correlated with the miR-20b expression (Fig. 2A and 6B), implicating the involvement of other factors/mechanisms in the expression of PTEN and BRCA1, in addition to miR-20b. Transcription factors, miRNAs and target proteins may form a complex network that plays an essential role in biologic and pathologic processes [50]. Although here we only showed that miR-20b transcriptionally activated by EGR1 directly targets PTEN and BRCA1, PTEN may also be directly activated by EGR1 [51], and BRCA1 may also play a role in the expression of other microRNAs, such as miR-155 [52].

To further establish the relationship between EGR1 and miR-20b expression in a large amount of breast tissue samples, we performed immunohistochemical and FISH analyses of EGR1 and miR-20b on breast cancer tissue arrays. Our data indicated that EGR1 expression was significantly correlated with miR-20b expression in normal, benign, and breast cancer tissues (Pearson Correlation r=0.99, Fig. 6A and B). Furthermore, both EGR1 and miR-20b were overexpressed in 50% metastatic breast cancer tissues compared with normal tissues and were correlated with each other remarkably well (Fig. 6C and D). These results further confirmed the relationship between EGR and miR-20b expression, as well as the involvement of miR-20b in the metastasis of breast cancer cells. Although normal tissues adjacent to tumors are generally used in comparative studies, it may be debateable whether or not the adjacent normal breast tissues used in these studies were really “normal”. A recent report indicated that the “normal” tissue adjacent to pancreatic cancer has already acquired a number of transcriptional alterations, and therefore is not an appropriate baseline for comparison with cancers[53]. This may partially explain why EGR1 and miR-20b are elevated 44% and 31%, respectively, in adjacent normal breast tissues examined (n=16). Although we did not analyze the correlation between miR-20b and triple-negative primary breast cancers due to the restriction in clinical data, other miRNAs, such as miR-21, miR-210 and miR-221 have been reported to play a significant role in these breast cancers[54].

In summary, the transcription factor EGR1 is a key regulator in the transcriptional control of miR-20b, which is aberrantly expressed in breast cancer tissues and cell lines. miR-20b may serve as an oncomiR that plays a crucial role in breast tumorigenesis by targeting tumor suppressors PTEN and BRCA1.

## MATERIALS AND METHODS

### Animal irradiation

Six-week old female Long Evans rats were randomly assigned to different treatment groups. Group 1: 30 kVp X-ray, 0.1 Gy (low dose/low energy, cumulative dose from multiple mammography screen); Group 2: 80 kVp X-ray, 2.5 Gy (High dose/high energy); and, Group 3: sham-treated controls. Ten rats per group were sacrificed at 6 hours, 96 hours, 4 weeks or 24 weeks after irradiation. Mammary gland specimens were frozen immediately and stored at −80°C, or fixed in 10% neutral buffered formalin and embedded in paraffin. Handling and care of animals were in accordance with the recommendations of the Canadian Council for Animal Care and Use. Procedures were approved by the University of Lethbridge Animal Welfare Committee. Animals were housed in a virus-free facility and given food and water *ad libitum*.

### microRNA profiling

Total RNA was isolated from mammary gland tissue of different group IR-exposed rats using TRIzol reagent (Invitrogen) according to the manufacturer's instruction. MicroRNA profiling, clustering and data analysis were carried out by LC Sciences.

### Cell culture

Human mammary epithelial cells (HMEC) purchased from Invitrogen were cultured in HuMEC Basal Serum-Free Medium (Invitrogen) containing HuMEC Supplement (Invitrogen); breast cancer cell lines ZR75-1, HCC1419, and HCC1806 obtained from ATCC were grown in ATCC-formulated RPMI-1640 Medium (ATCC) containing 10% FBS; MCF7 cells were cultured in DMEM/F-12 (HyClone) containing 10% FBS; HEK293 cells were grown in DMEM/High Glucose (Thermo Scientific Limited) containing 10% FBS at 37°C in a humidified atmosphere of 5% CO_2_.

### microRNA real-time RT-PCR

Total RNAs isolated from IR-exposed rat mammary gland tissue, HMEC, MCF7, ZR75-1, HCC1419, and HCC1806 cells were subjected to real-time RT-PCR using primer sets for either rno-miR-20b-5p or hsa-miR-20b (SABiosciences) according to the manufacturer's instruction. Rat and human RNU6-2 served as a loading control.

### miR-20b gene copy number analysis

Genomic DNAs extracted from IR-exposed HMEC cells using a kit for purification of total DNA from animal blood or cells (QIAGEN) were subjected to real-time PCR using SsoFast EvaGreen Spermix (Bio-Rad) with the following primers; 20bCopyNo-F: 5'-TGC AGG TAG TTT TGG CAT GA-3', 20bCopyNo-R: 5'-TCA ACA AGA GAT TTG TTA TCC AAG AG-3'; RPP38-F: 5'-TGG TTG TGA AGA CGT CGT TGA-3', RPP38-R: 5'-TGC ATA TCC TCG CTC TCC AGA-3'. The copy number level relative to the internal control (RNase P/*RPP38*) was calculated by the comparative threshold cycle (Ct) method, and results are shown as fold induction.

### Immunofluorescence

HMEC cells grown on coverslips were exposed to either 30 kVp/0.1 Gy, 80 kVp/2.5 Gy X-ray or left sham-treatment as a control. 96 hours after irradiation, immunofluorescence staining was performed using rabbit monoclonal antibody against EGR1 (Cell Signaling Technology) as described previously.^55^ Fluorescence was observed under 400x on an inverted microscope (ZEISS).

### EGR1 real-time RT-PCR

Total RNA isolated from HCC1806 transiently transfected with 10 nM or 50 nM of siEGR1 (QIAGEN) or 50 nM of AllStar negative control (QIAGEN) for 96 hours was subjected to real-time RT-PCR using EGR1 primers (QuantiTect Primer Assay, QIAGEN) and SsoFast EvaGreen Supermix (BIO-RAD) according to manufacturer's instruction.

### Generation of plasmid constructs

Wild-type and mutant fragments of the hsa-miR-20b promoter amplified by PCR using genomic DNA were cloned into pGEM-T easy vector (Promega), released by digestion with *Kpn* I and *Hind* III, and subcloned into pGL3-Basic vector (Promega); sequence identity was confirmed by automatic sequencing; primers used here for amplifying miR-20b promoters are as follows, 20b WT-Prom F: 5'-ATT GGT ACC GTT TTC GCT TTG-3', 20b mt-Prom F: 5'-TTG GTA CCG AGA CTG CGC T-3', 20b Prom R: 5'-ATA AGC TTG CCC CAA CGA AG-3'. To generate luciferase miR-20b target reporters, oligos corresponding to portions of the 3'UTRs of either PTEN or BRCA1 were synthesized, annealed and cloned into downstream of the luciferase gene in the pGL3-Basic vector between *Xba* I and *Eco*R I (a linker introduced by Dr James Meservy); the sequence identity was confirmed by automatic sequencing. Oligo sequences were as follows. PTEN 3'UTR-1: 5'-/5Phos/CTA GAA GAT GGC ACT TTC ACT GCT TGT TGT TTG CGC ATT TTT G-3', PTEN 3'UTR-2: 5'-/5Phos/AAT TCA AAA ATG CGC AAA CAA CAA GCA GTG AAA GTG CCA TCT T-3'; BRCA1 3'UTR-1: 5'-/5Phos/CTA GAT CAC GCC TGT AAT CCC AGC ACT TTG GGA G-3', BRCA1 3'UTR-2: 5'-/5Phos/AAT TCT CCC AAA GTG CTG GGA TTA CAG GCG TGA T-3'.

### Bioinformatics

The transcription start site of hsa-miR-20b was predicted using Promoter 2.0 Prediction Server. Common transcription factor binding sites at hsa-miR-20b promoter were analysed using Genomatix. Potential hsa-miR-20b targets were predicted by both MIRANDA and RNAhybrid softwares. A network of predicted hsa-miR-20b targets were generated by STRING 9.0.

### Cell cycle and apoptosis analyses

HCC1806 cells grown to 90% confluency (as determined by microscopy analysis) were transiently transfected with either miRCURY LNA hsa-miR-20b power inhibitor or miRCURY LNA microRNA power inhibitor negative control A (Exiqon). 96 hours after transfection, the cells were harvested for cell cycle and apoptosis analyses that were performed with a BD FACSCanto II Flow Cytometer (BD Biosciences) using propidium iodide staining solution and FITC Annexin V Apoptosis Detection Kit II (BD Biosciences) according to manufacturer's instruction.

### Western blot analysis

HMEC, MCF7, ZR75-1, HCC1419, and HCC1806 cells grown to 90% confluency were rinsed twice with ice-cold PBS and scraped off the plate in radioimmunoprecipitation assay buffer (RIPA). 30-100 μg of protein per sample was electrophoresed on 6% or 10% SDS-PAGE and electrophoretically transferred to PVDF membrane (Amersham Hybond^™^-P, GE Healthcare) at 4°C for 1.5 hours. Blots were incubated for 1 hour with 5% nonfat dry milk to block nonspecific binding sites and then incubated with polyclonal/monoclonal antibodies against PTEN, BRCA1 (Santa Cruz Biotechnology) or EGR1 (Cell Signaling Technology) at 4°C overnight. The immunoreactivity was detected using peroxidase-conjugated antibody and visualized by ECL Plus Western Blotting Detection System (GE Healthcare). The blots were stripped before reprobing with antibodies to GAPDH or actin (Santa Cruz Biotechnology).

### MTT assay

24 hours after transfection with miR-20b inhibitor (Exiqon), 3.0 × 10^3^ HCC1806 cells were plated in 96-well plates. 3-(4,5-Dimethylthiazol-2-yl)-2,5-diphenyltetrazolium bromide assays were carried out using the Cell Proliferation Kit I (Roche Diagnostics GmbH) as described by the manufacturer. The spectrophotometric absorbance of samples was measured at 595 nm using a microtiter plate reader (FLUOstar Omega, BMG LABTECH).

### Wound healing assay

24 hours after transfection with miR-20b inhibitor (Exiqon), HCC1806 cells were replated in 6-well plates and incubated at 37°C in a humidified atmosphere of 5% CO_2_ for another 24 hours. The cells were treated with 10 μg/ml mitomycin C (Sigma) for 2 hours before injury. The wound healing assay was carried out as described previously[56].

### Transient transfection and luciferase assay

HEK293 cells grown to 90% confluence in 6-well plates in antibiotic-free DMEM/High Glucose (Thermo Scientific Limited) containing 10% FBS were transiently cotransfected with either 0.5 μg WT-miR20b promoter or MT-miR20b promoter reporter, 0.2 μg or 1 μg pCB6-Egr1, 5 ng pRL-TK, and left empty vector pCB6 as a control; or contransfected with 0.4 μg reporter plasmid (either pGL3-PTEN or pGL3-BRCA1), 5 ng pRL-TK plasmid, 10 nM or 50 nM hsa-miR-20b mimic (5'-CAA AGU GCU CAU AGU GCA GGU AG-3', QIAGEN) and mirVana miRNA mimic negative control #1 (Ambion) using Lipofectamine 2000 (Invitrogen, Carlsbad, CA, USA) as per manufacturer's instruction. 24 hours after transfection, cells were harvested, the relative luciferase activity was measured by the Dual-Luciferase Reporter Assay System (Promega) using a luminometer (FLUOstar Omega, BMG LABTECH) and with *Firefly* luciferase data normalized to *Renilla* luciferase.

### ChIP-PCR

HMEC and HCC1806 cells grown to 90% confluence were subjected to quantitative ChIP assays as detailed elsewhere[57, 58]. Briefly cells were treated with 0.4% formaldehyde and the cross-linked chromatin retrieved by nuclei isolation and lysis. The chromatin was sonicated to ~300 bp, pre-cleared with rabbit serum and immunoprecipitated with ChIP-grade rabbit monoclonal antibody to EGR1 (Cell Signaling Technology). Enrichments were measured by both conventional PCR and real-time PCR using SsoFast EvaGreen Supermix (Bio-Rad) as previously described [58]. The levels of enrichment were normalized to that obtained with total input. The following primer pairs were used; Hu20b-EGR1-ChIP-PCR F: 5'-GGA AGA GAG AAG GGC TTT GG-3', HU20B-EGR1-CHIP-PCR R: 5'-TGC CTT TAA TAG CCC AAG GA-3'.

### Electrophoretic mobility shift assay (EMSA)

HCC1806 cells grown to 90% confluency, nuclear extracts were prepared using NE-PER Nulcear and Cytoplasmic Extraction Reagents (Thermo Scientific Limited), and EMSA was performed using Lightshift Chemilluminescent EMSA Kit (Thermo Scientific Limited) according to the manufacturer's instruction. 20 μl of binding reaction contained 1 × EGR1 binding buffer, 50 ng/μl Poly(dI/dC), 0.05% NP-40, 800 nM cold probe (20bEGR1-EMSA-Oligo 1: 5'-GGC CGG GTG GGC GGG GGC GGG C-3; 20bEGR1-EMSA-Oligo 2: 5'-GCC CGC CCC CGC CCA CCC GGC C-3'), 2 nM Biotin probe (20bEGR1-EMSA-Biotin 1: 5'-Biotin/GGC CGG GTG GGC GGG GGC GGG C-3'; 20bEGR1-EMSA-Biotin 2: 5'-Biotin/ GCC CGC CCC CGC CCA CCC GGC C-3'), 2 μl nuclear extract, 1 μg/μl BSA, 2 μl EGR1 antibody or 2 μl normal rabbit IgG (Cell Signaling Technology).

### Fluorescence in situ hybridization (FISH)

Hsa-miR-20b expression in breast cancer specimens (BRC961, BRC962 and BRM961 arrays; Pantomics) was determined by FISH as detailed elsewhere [59]. Briefly, after deparafinization, the sections were prehybidized for 20 minutes at 55°C, followed by 1 hour hybridization at the same temperature with 1:1000 dilution of miRCURY LNA hsa-miR-20b detection probe (Exiqon); after washing, the sections were blocked for 1 hour with blocking solution, and incubated with 1:1000 dilution of anti-Digoxigenin-Fluorescein, Fab fragments (Roche) at 4°C overnight.

### RNA pulldown and RNA sequencing analysis

Total RNA isolated from HCC1806 cells using TRIzol Reagent (Invitrogen) and treated RNase-free DNase I (Fermentas) was subjected to RNA pulldown assay using μMACS Streptavidin Kit (Miltenyi Biotec Inc.) as described previously [60] and according to the manufacturer's instruction. 15 μg of total RNA and 1 μg of biotinylated capture DNA were used in the RNA pulldown, including wild-type miR-20b capture oligo: 5Biosg/CAA AGT GCT CAT AGT GCA GGT AG, and scrambled miR-20b capture oligo: 5Biosg/CCA GTG AAT CAT AGT GCA GGT AG (Exiqon). 300 ng of total pulldown RNA was subjected to high throughput RNA sequencing analysis using our in house sequencing platform (Illumina, Genome Analyser). Sequences with >2-fold increase compared to scrambled oligo were pulled out for further analysis. Prediction of miR-20b-targeting mRNAs was performed using MIRANDA and RNAhybrid with default settings[61, 62]. A network of the predicted tumor suppressors targeted by miR-20b was generated by STRING 9.0.

### Immunohistochemical analysis

The expression of EGR1, PTEN and BRCA1 in breast cancer specimens (BRC961, BRC962 and BRM961 arrays; Pantomics) was determined by immunohistochemical staining using antibodies to EGR1 (Cell Signaling Technology), PTEN and BRCA1 (Biocare Medical, performed by Pantomics) as described by manufacturers. Stained tissue sections were analyzed independently by a pathologist and scientists in blind manner.

### Statistical analysis

The Student's *t* test was used for statistical significance of differences in miR-20b expression, EGR1 expression, luciferase activity, enrichment of EGR1 at miR-20b promoter, cell growth, and cell migration between groups. Pearson Correlation was used for statistical significance between miR-20b expression and the exression of EGR1 or PTEN or BRCA1 in breast cancer specimens examined. *P* < 0.05 was considered significant.

### Disclosure of Potential Conflicts of Interest

No potential conflicts of interest were disclosed.

## Supplementary Figures


