# Supplementary material for: Crucial Role for Early Growth Response-1 in the Transcriptional Regulation of miR-20b in Breast Cancer
Source: Oncotarget. 2013 Jul 28;4(9):1373–87. doi: 10.18632/oncotarget.1165 (PMC3824527; doi:10.18632/oncotarget.1165)
Supplement: Supplementary file 1 [file oncotarget-04-1373-s001.pdf]

Crucial Role for Early Growth Response-1 in the Transcriptional Regulation of miR-20b in Breast Cancer - Li et al

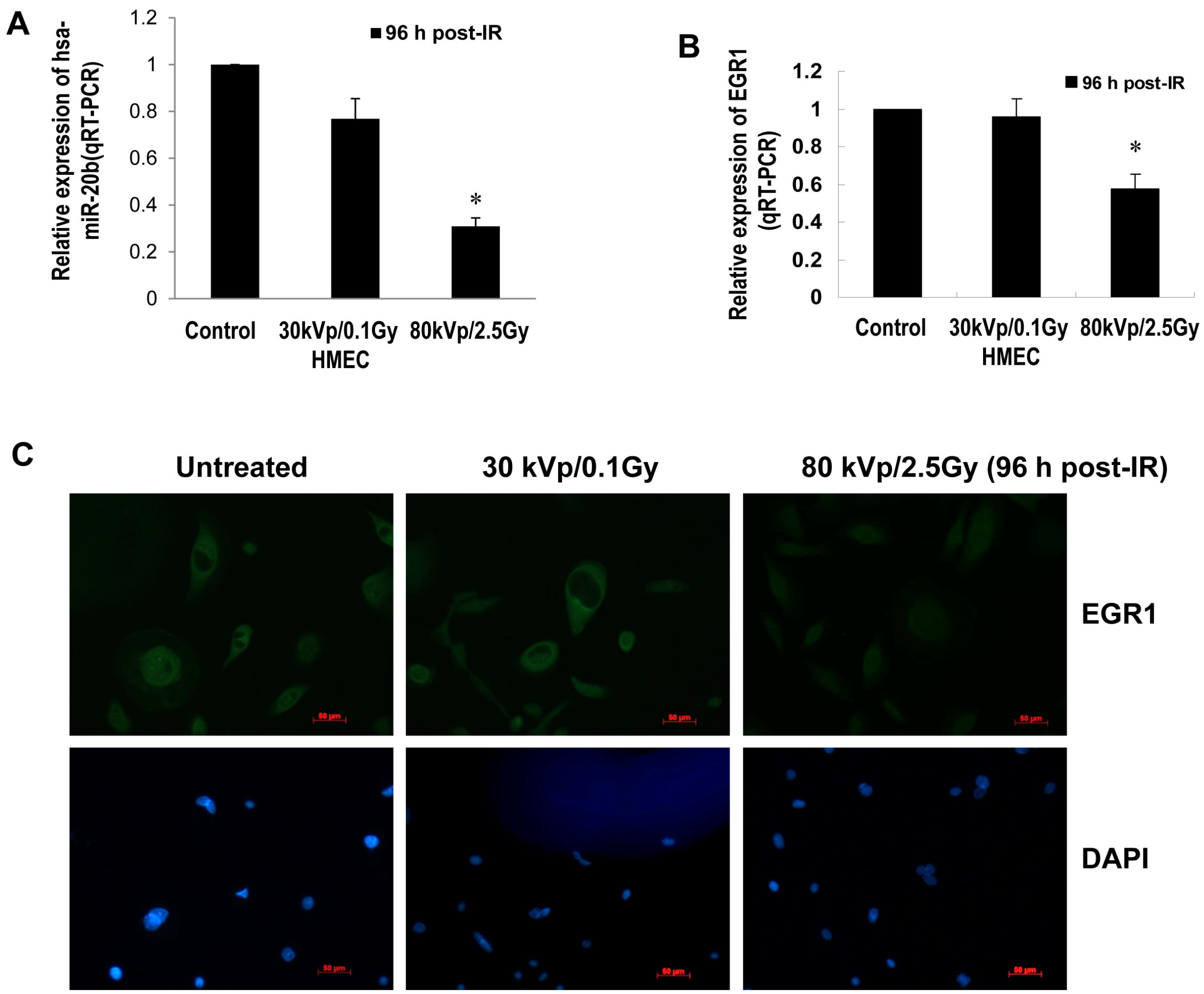

Figure S1: Downregulation of hsa-miR-20b and EGR1 triggered by IR. (A and B) 96 hours post-IR, total RNA isolated from HMEC exposed to either 30 kVp/0.1 Gy or 80 kVp/2.5 Gy X-ray was subjected to real-time RT-PCR using primers for hsa-miR-20b and EGR1. (C) HMEC cells grown on coverslips were exposed to either 30 kVp/0.1 Gy, 80 kVp/2.5 Gy X-ray, or left sham-treatment as a control; 96 hours after irradiation, immunofluorescence staining was performed using rabbit monoclonal antibody against EGR1. The asterisk indicates $p<0.05$.

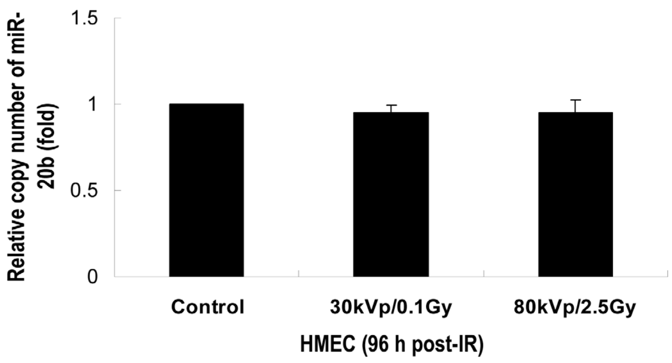

Figure S2: miR-20b gene copy number may not contribute to IR-induced miR-20b expression. Genomic DNA was extracted from IR-exposed HMEC cells, and the copy number for miR-20b gene was determined.

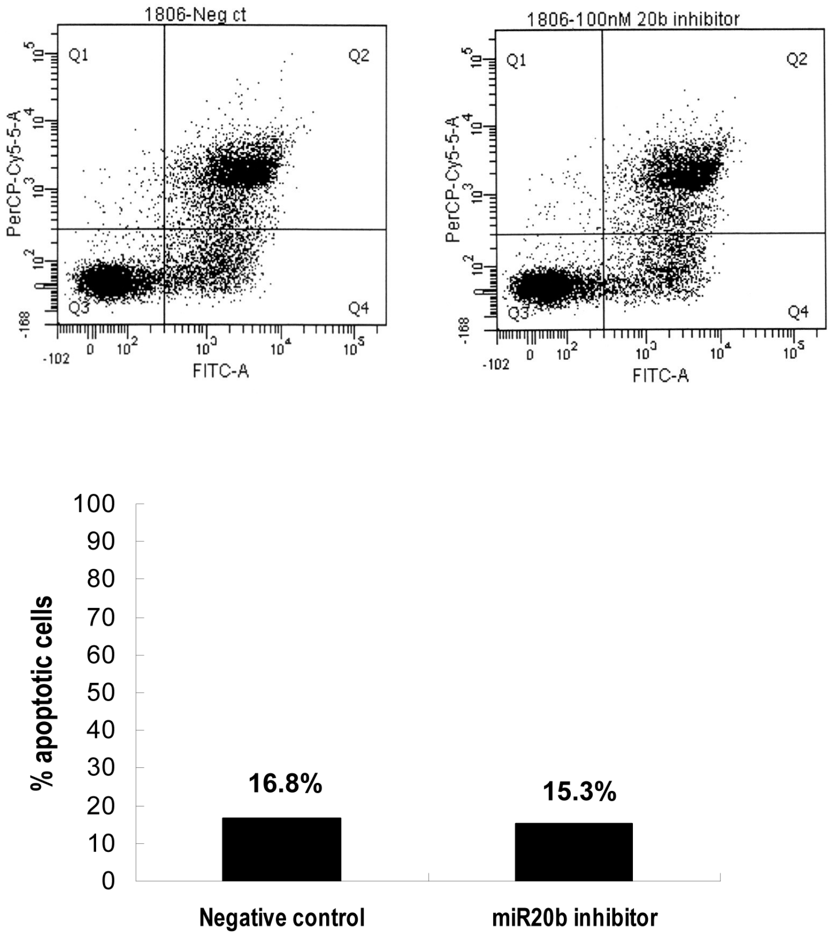

Figure S3: Hsa-miR-20b does not affect apoptosis of breast cancer cells. HCC1806 cells grown to 90% confluency were transfected with either hsa-miR-20b inhibitor or negative control A; 96 hours after transfection, the cells were harvested for apoptosis analysis using FITC Annexin V Apoptosis Detection Kit II (BD Biosciences) according to the manufacturer's instruction.

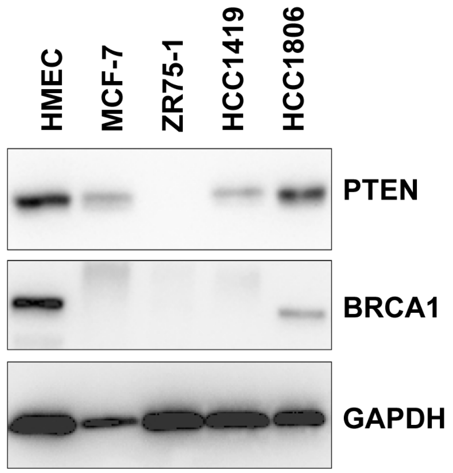

Figure S4: Downregulation of PTEN and BRCA1 in breast cancer cell lines. Whole cellular lysates prepared from HMEC, MCF7, ZR75-1, HCC1419, and HCC1806 cell lines were subjected to Western blot analysis using antibodies specific to PTEN and BRCA1.

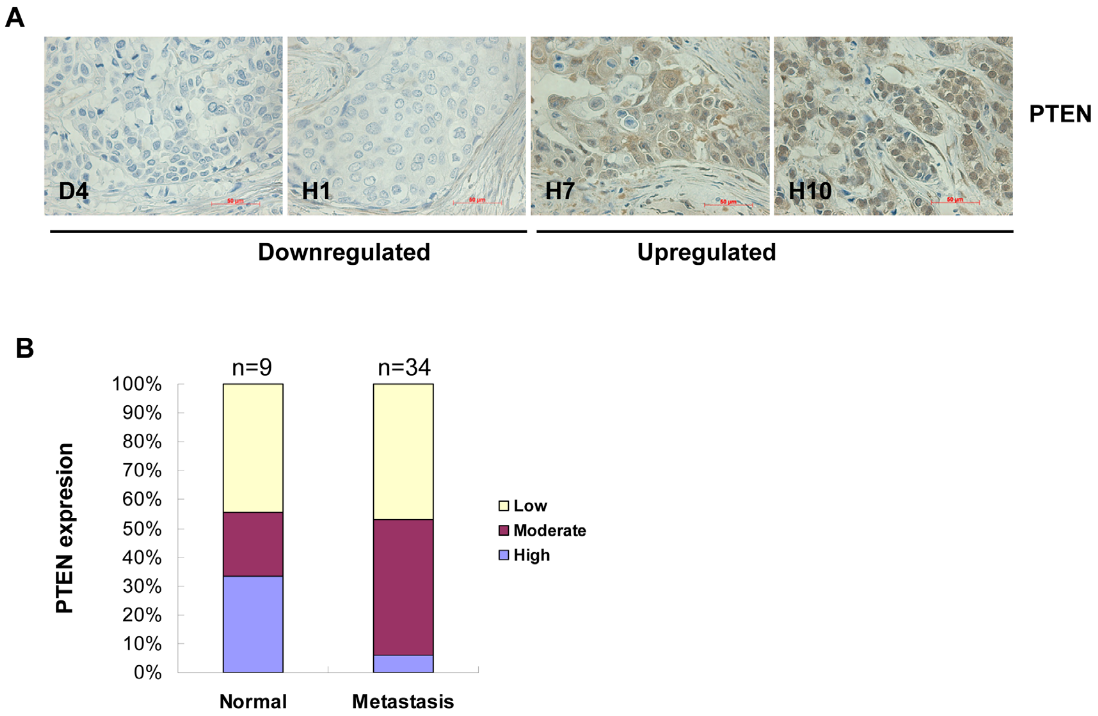

Figure S5: The expression of PTEN in metastatic breast cancer tissues. (A) representatives of PTEN staining in the sections of metastatic breast cancer tissue array. (B) statistical analysis of PTEN expression in the metastatic breast cancer tissues.

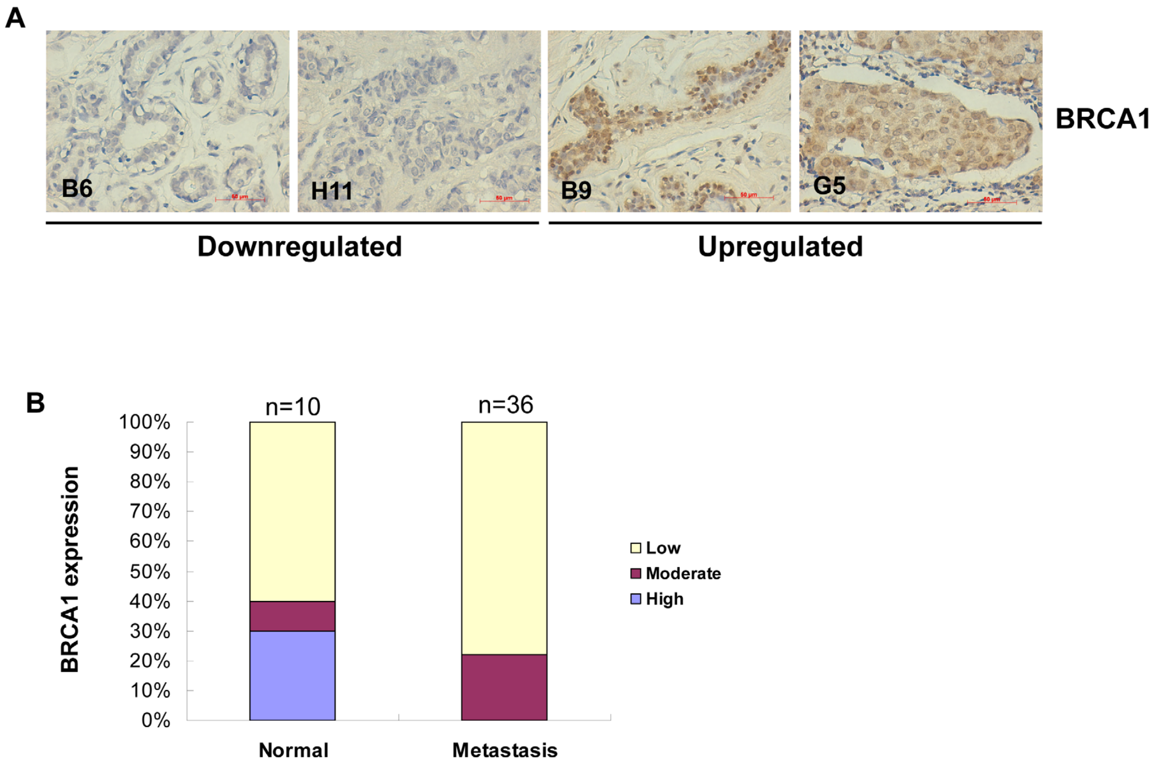

Figure S6: The expression of BRCA1 in metastatic breast cancer tissues. (A) representatives of BRCA1 staining in the sections of metastatic breast cancer tissue array. (B) statistical analysis of BRCA1 expression in the metastatic breast cancer tissues.

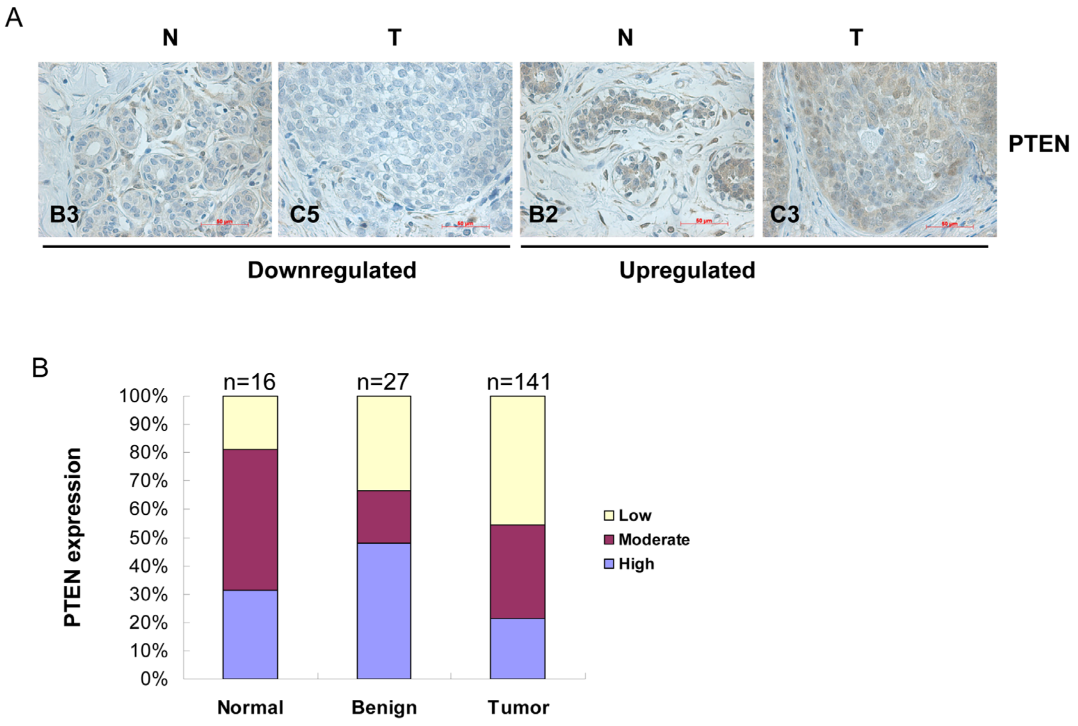

Figure S7: The expression of PTEN in breast cancer tissues. (A) representatives of PTEN staining in the sections of breast cancer tissue array. (B) statistical analysis of PTEN expression in the metastatic breast cancer tissues.

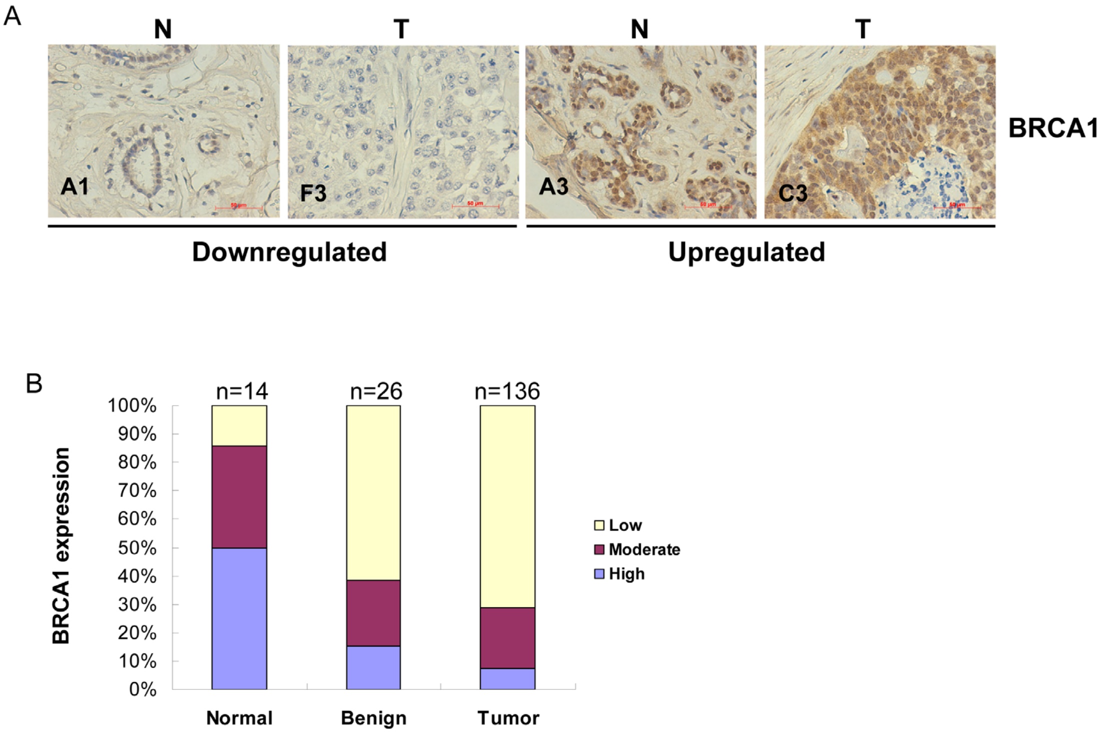

Figure S8: The expression of BRCA1 in breast cancer tissues. (A) representatives of BRCA1 staining in the sections of breast cancer tissue array. (B) statistical analysis of BRCA1 expression in the breast cancer tissues.
